# Identification of Oxygen-Independent Pathways for Pyridine Nucleotide and Coenzyme A Synthesis in Anaerobic Fungi by Expression of Candidate Genes in Yeast

**DOI:** 10.1128/mBio.00967-21

**Published:** 2021-06-22

**Authors:** Thomas Perli, Aurin M. Vos, Jonna Bouwknegt, Wijb J. C. Dekker, Sanne J. Wiersma, Christiaan Mooiman, Raúl A. Ortiz-Merino, Jean-Marc Daran, Jack T. Pronk

**Affiliations:** a Department of Biotechnology, Delft University of Technology, Delft, The Netherlands; Universite Nice Sophia Antipolis; Tel Aviv University

**Keywords:** Neocallimastigomycetes, *Saccharomyces cerevisiae*, anaerobes, biotechnology, fungi, nicotinic acid, oxygen requirement, pantothenate, vitamin biosynthesis

## Abstract

Neocallimastigomycetes are unique examples of strictly anaerobic eukaryotes. This study investigates how these anaerobic fungi bypass reactions involved in synthesis of pyridine nucleotide cofactors and coenzyme A that, in canonical fungal pathways, require molecular oxygen. Analysis of Neocallimastigomycetes proteomes identified a candidate l-aspartate-decarboxylase (AdcA) and l-aspartate oxidase (NadB) and quinolinate synthase (NadA), constituting putative oxygen-independent bypasses for coenzyme A synthesis and pyridine nucleotide cofactor synthesis. The corresponding gene sequences indicated acquisition by ancient horizontal gene transfer (HGT) events involving bacterial donors. To test whether these enzymes suffice to bypass corresponding oxygen-requiring reactions, they were introduced into *fms1*Δ and *bna2*Δ Saccharomyces cerevisiae strains. Expression of *nadA* and *nadB* from Piromyces finnis and *adcA* from Neocallimastix californiae conferred cofactor prototrophy under aerobic and anaerobic conditions. This study simulates how HGT can drive eukaryotic adaptation to anaerobiosis and provides a basis for elimination of auxotrophic requirements in anaerobic industrial applications of yeasts and fungi.

## INTRODUCTION

Neocallimastigomycetes are obligately anaerobic fungi with specialized metabolic adaptations that allow them to play a key role in the degradation of recalcitrant plant biomass in herbivore guts (1). Despite complicated cultivation techniques and lack of genetic modification tools (2), several evolutionary adaptations of these eukaryotes to an anaerobic lifestyle have been inferred from biochemical studies (3–5). Sequence analysis implicated extensive horizontal gene transfer (HGT) as a key mechanism in these adaptations (6–8). For example, instead of sterols, which occur in membranes of virtually all other eukaryotes (9) and whose biosynthesis involves multiple oxygen-dependent reactions (10), Neocallimastigomycetes contain tetrahymanol (3, 6). This sterol surrogate (11) can be formed from squalene by a squalene:tetrahymanol cyclase (STC), whose structural gene in Neocallimastigomycetes showed evidence of acquisition by HGT from prokaryotes (6, 12). Expression of an STC gene was recently shown to enable sterol-independent anaerobic growth of the model eukaryote Saccharomyces cerevisiae (13).

Further exploration of oxygen-independent bypasses in Neocallimastigomycetes for intracellular reactions that in other eukaryotes require oxygen is relevant for a fundamental understanding of the requirements for anaerobic growth of eukaryotes. In addition, it may contribute to the elimination of nutritional requirements in industrial anaerobic applications of yeasts and fungi.

**FIG 1:**
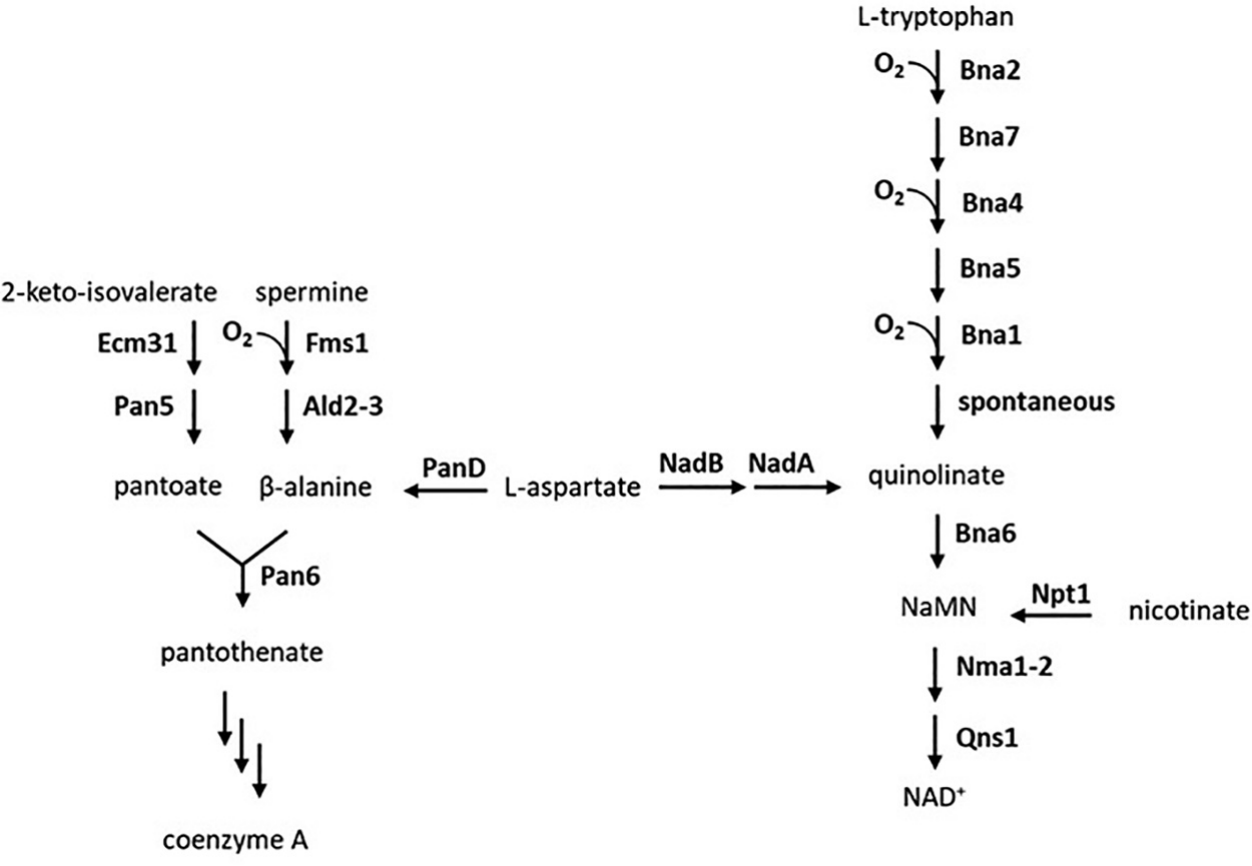
CoA and NAD^+^ biosynthetic pathways in S. cerevisiae and oxygen-independent alternatives. CoA synthesis includes the condensation of pantoate and β-alanine. (Left) In S. cerevisiae, β-alanine is formed from spermine in two steps using the oxygen-dependent polyamine oxidase Fms1. Other organisms, including Archaea, Bacteria, and insects, can bypass this oxygen requirement by synthesizing β-alanine from aspartate using l-aspartate decarboxylase (AdcA/PanD). (Right) NAD^+^ is synthesized via the kynurenine pathway in 9 reactions starting from tryptophan, 3 of which require oxygen. Other organisms that include plants and bacteria are able to bypass this oxygen requirement by synthesizing quinolinate from aspartate using l-aspartate oxidase and quinolinate synthase (NadB and NadA, respectively).

Most fungi are capable of *de novo* synthesis of pyridine nucleotide cofactors (NAD^+^ and NADP^+^) and coenzyme A (CoA) when grown aerobically. As exemplified by the facultatively anaerobic yeast S. cerevisiae (14), canonical fungal pathways for synthesis of these cofactors are oxygen dependent. In S. cerevisiae, biosynthesis of CoA involves formation of β-alanine by the oxygen-requiring polyamine oxidase Fms1 (15). This intermediate is then condensed with pantoate to yield the CoA precursor pantothenate (16, 17) (Fig. 1, left). Similarly, the yeast kynurenine pathway for *de novo* synthesis of NAD^+^ involves three oxygen-dependent reactions, catalyzed by indoleamine 2,3-dioxygenase (Bna2; EC 1.13.11.52), kynurenine 3-monooxygenase (Bna4; EC 1.14.13.9), and 3-hydroxyanthranilic-acid dioxygenase (Bna1; EC 1.13.11.6) (14) (Fig. 1, right). The Neocallimastigomycetae Neocallimastix patriciarum has been shown to grow in synthetic media lacking precursors for pyridine nucleotide and CoA synthesis (18). This observation indicates that at least some anaerobic fungi harbor oxygen-independent pathways for synthesizing these essential cofactors. Genomes of Neocallimastigomycetes lack clear homologs of genes encoding the oxygen-requiring enzymes of the kynurenine pathway. Instead, their genomes were reported to harbor genes encoding an l-aspartate oxidase (NadB) and quinolinate synthase (NadA), two enzymes active in the bacterial pathway for NAD^+^ synthesis (6) (Fig. 1, right). Since bacterial and plant aspartate oxidases can, in addition to oxygen, also use fumarate as electron acceptor (19, 20), it is conceivable that NadA and NadB may allow for oxygen-independent NAD^+^ synthesis in anaerobic fungi. No hypothesis has yet been forwarded on how these fungi may bypass the oxygen requirement for the canonical fungal CoA biosynthesis route.

The goals of this study were to identify the pathway responsible for oxygen-independent synthesis of CoA in Neocallimastigomycetes and to investigate a possible role of NadA and NadB in oxygen-independent synthesis of pyridine nucleotide cofactors. A candidate l-aspartate decarboxylase (Adc)-encoding gene was identified by genome analysis of Neocallimastigomycetes, and its phylogeny was investigated. Candidate Neocallimastigomycetes genes for l-aspartate oxidase and quinolinate synthase, previously reported to have been acquired by HGT (6), as well as the candidate Adc gene, were then functionally analyzed by expression in S. cerevisiae strains devoid of essential steps in the native cofactor synthesis pathways. As controls, previously characterized genes involved in oxygen-independent NAD^+^ biosynthesis by Arabidopsis thaliana (21) and a previously characterized Adc-encoding gene from the red flour beetle Tribolium castaneum (*TcPAND*) (22) were also expressed in the same S. cerevisiae strains. The results demonstrate how heterologous expression studies in yeast can provide insight into evolutionary adaptations to anaerobic growth and selective advantages conferred by proposed HGT events in Neocallimastigomycetes. In addition, they identify metabolic engineering strategies for eliminating oxygen requirements for cofactor biosynthesis in anaerobic industrial applications of S. cerevisiae.

## RESULTS

### Identification of a candidate oxygen-independent l-aspartate decarboxylase involved in CoA synthesis in anaerobic fungi.

Decarboxylation of l-aspartate to β-alanine by l-aspartate decarboxylase (Adc), an enzyme that occurs in many species across all domains of life (23), enables an oxygen-independent alternative for the canonical fungal pathway for CoA synthesis (Fig. 1). To explore its occurrence in anaerobic fungi, a set of 51 amino acid sequences of Adc homologs listed by Tomita et al. (23) were used as queries against all proteins from 5 Neocallimastigomycetes species deposited in the TrEMBL section of the UniProt database. This search yielded 16 Neocallimastigomycetes hits (E value < 10^−6^) (see Table S1 in the supplemental material), six of which originated from Neocallimastix californiae. Only one of these hits, A0A1Y1ZL74, did not reveal annotation errors upon transcriptome sequencing (RNA-seq) read mapping, showed the highest read coverage (see Fig. S1), and was selected as the best Neocallimastigomycetes Adc candidate.

10.1128/mBio.00967-21.1 TABLE S1 Neocallimastigomycetes BLASTP hits. Obtained using the dataset from Tomita et al. (23) as queries against a Neocallimastigomycetes-specific amino acid sequence database and an E value of <10^−6^ as the cutoff. Download Table S1, DOCX file, 0.02 MB. Copyright © 2021 Perli et al. 2021 Perli et al. https://creativecommons.org/licenses/by/4.0/ This content is distributed under the terms of the Creative Commons Attribution 4.0 International license.

10.1128/mBio.00967-21.2 FIG S1 Verification of l-aspartate decarboxylases from *Neocallimastix californiae* using RNA-seq data. Illumina libraries were obtained from the Sequence Read Archive using the SRR7140690 run identifier. Reads were mapped using STAR 2.6.1a_08-27 against genome assembly GCA_002104975. Alignments were processed using SAMtools 1.3.1 and visualized using Artemis. Errors are highlighted inside black dashed lines. (A) A0A1Y2ADH1; (B) A0A1Y2B2H7; (C) A0A1Y2AA19; (D) A0A1Y1ZL74. Download FIG S1, TIF file, 0.4 MB. Copyright © 2021 Perli et al. 2021 Perli et al. https://creativecommons.org/licenses/by/4.0/ This content is distributed under the terms of the Creative Commons Attribution 4.0 International license.

**FIG 2:**
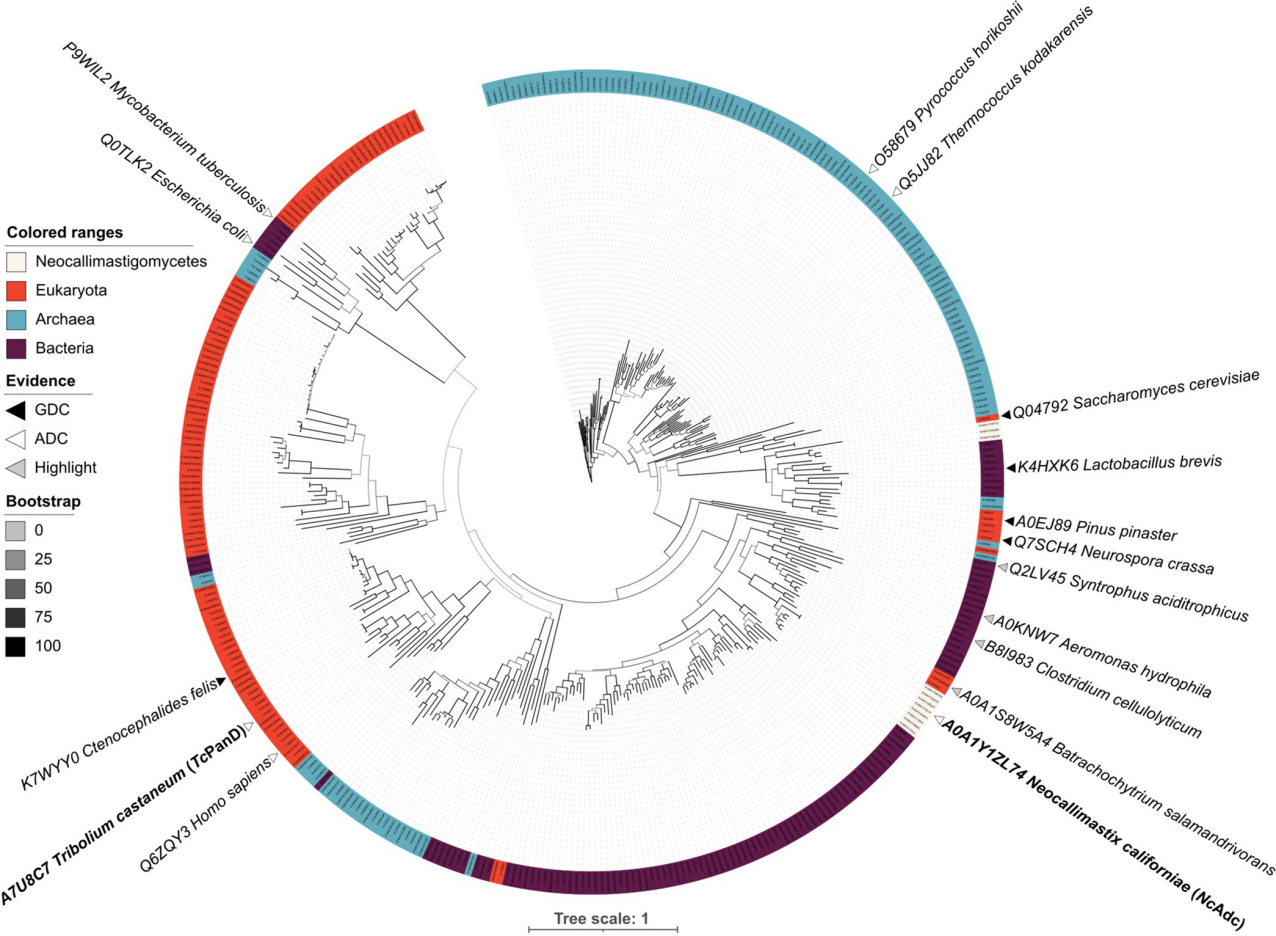
Unrooted maximum likelihood phylogenetic tree of aspartate decarboxylase and glutamate decarboxylase homologs. Sequences of proteins with demonstrated enzyme activity are marked with white triangles (l-aspartate decarboxylases) or black triangles (glutamate decarboxylases). Interactive visualizations with all sequence identifiers, branch distances, and bootstrap values can be accessed at https://itol.embl.de/tree/838448017961605604402 and https://itol.embl.de/tree/8384480476641615985323.

The amino acid sequence A0A1Y1ZL74 (here referred to as *Nc*AdcA) was used for a second round of homology search to obtain a broad set of Adc-like sequences, with a similar sequence representation of taxa across the three domains of life (104 sequences from Bacteria, 101 from Eukarya, and 120 from Archaea) (see Data Set S1). The complete set of *Nc*AdcA homologs (together with the set defined by Tomita et al. [23] and their Neocallimastigomycetes homologs) (see Data Set S2) was subjected to multiple-sequence alignment. A subsequent phylogenetic tree (Fig. 2; Data Set S3) showed that *Nc*Adc sequences are closely related to those of chytrid fungi (e.g., A0A1S8W5A4 from Batrachochytrium salamandrivorans) and anaerobic bacteria (e.g., B8I983 from Clostridium cellulolyticum, currently known as Ruminiclostridium cellulolyticum [24]; we used the former name for consistency with UniProt identifiers). These Neocallimastigomycetes, chytrid, and bacterial Adc homologs were more closely related to each other than to characterized eukaryotic Adc and bacterial PanD sequence*s.* Furthermore, HMMER E values obtained from using *Nc*AdcA as the query against the bacterial database were more significant than when using the eukaryotic or archaeal databases (see Fig. S2; Data Set S1). These results suggest that a bacterial ancestor donated an Adc-encoding sequence to a common ancestor of chytrids and Neocallimastigomycetes.

10.1128/mBio.00967-21.3 FIG S2 Distribution of HMMER E values for *Nc*AdcA (A0A1Y1ZL74) hits. Bars are colored as shown in Fig. 2. Values are presented in Data Set S1. Download FIG S2, TIF file, 0.2 MB. Copyright © 2021 Perli et al. 2021 Perli et al. https://creativecommons.org/licenses/by/4.0/ This content is distributed under the terms of the Creative Commons Attribution 4.0 International license.

10.1128/mBio.00967-21.4 DATA SET S1 HMMER E values for *Nc*AdcA (A0A1Y1ZL74) hits. Download Data Set S1, XLSX file, 0.03 MB. Copyright © 2021 Perli et al. 2021 Perli et al. https://creativecommons.org/licenses/by/4.0/ This content is distributed under the terms of the Creative Commons Attribution 4.0 International license.

**FIG 3:**
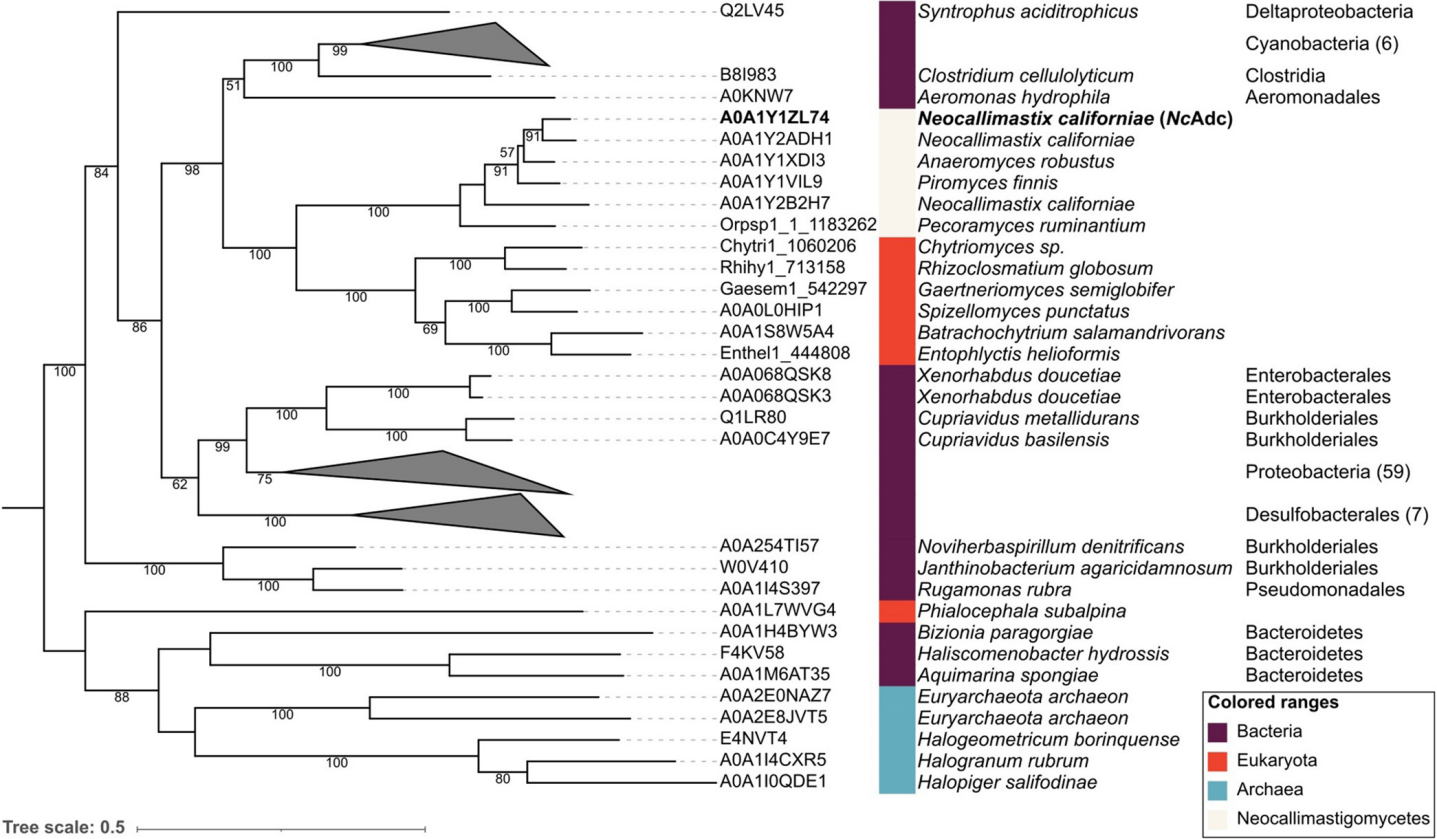
Mid-rooted maximum likelihood phylogenetic tree of aspartate decarboxylase orthologs. Number of sequences in collapsed clades are indicated in parentheses. A summary of the search from which these sequences were obtained is presented in Table 1. An interactive visualization with all sequence identifiers, branch support, distances, and bootstrap values can be accessed at https://itol.embl.de/tree/8384480267191615280152.

10.1128/mBio.00967-21.5 DATA SET S2 (A) Three hundred eighty-seven amino acid sequences used for the phylogenetic analysis shown in Fig. 2. File provided in FASTA format with UniProt identifiers in the headers. Subscripts were added to the headers as follows: A_ for sequences from Archaea, B_ from *Bacteria*, E_ from Eukaryota, N_ from Neocallimastigomycetes, and T_ from Tomita et al. (23). (B) One hundred three amino acid sequences used for the phylogenetic analysis shown in Fig. 3. File provided in FASTA format Download Data Set S2, TXT file, 0.3 MB. Copyright © 2021 Perli et al. 2021 Perli et al. https://creativecommons.org/licenses/by/4.0/ This content is distributed under the terms of the Creative Commons Attribution 4.0 International license.

10.1128/mBio.00967-21.6 DATA SET S3 Raw phylogenetic tree used in Fig. 2. File provided in Newick format with UniProt identifiers in the headers labeled as indicated in Data Set S2. Download Data Set S3, TXT file, 0.01 MB. Copyright © 2021 Perli et al. 2021 Perli et al. https://creativecommons.org/licenses/by/4.0/ This content is distributed under the terms of the Creative Commons Attribution 4.0 International license.

To further investigate the potential bacterium-to-chytrid HGT event, a refined ortholog search and phylogenetic analysis were performed. Full proteomes of all species showing an *Nc*AdcA homolog, in addition to predicted proteomes from six chytrids used in a previous phylogenomic analysis (8), were retrieved and used to obtain all possible co-ortholog groups. From a total number of 103 *Nc*AdcA orthologs obtained, 85 were bacterial, 5 were archaeal, and 13 were eukaryotic (Table 1; Data Set S2). Eukaryotic *Nc*AdcA orthologs were only found in fungi, and 12 of 13 were found in species from the Chytridiomycota phylum. The latter included five of the six chytrids analyzed in the phylogenomic study by Wang et al. (8) and all Neocallimastigomycetes considered in this study. Further phylogenetic analysis of the 103 *Nc*AdcA orthologs indicated a common origin for bacterial and chytrid *Nc*AdcA (Fig. 3; Data Set S4). The closest bacterial relatives to *Nc*AdcA were found in the facultative anaerobe and waterborne bacterium Aeromonas hydrophila subsp. *hydrophila* ATCC 7966^T^ (25) and the ruminal anaerobe *C. cellulolyticum* strain H10 (24, 26). Additional close bacterial relatives were also strict anaerobes, such as the syntrophic bacterium Syntrophus aciditrophicus (27) and members of the *Desulfobacteraceae* family (28).

**TABLE 1:** Summary of *Nc*Adc homology search results across domains of life

Taxonomic rank	No. of species analyzed	No. of homologs	No. of orthologs
Eukarya	749	101	13
Fungi	404	48	13
Dikarya	372	36	1
Ascomycota	280	36	1
Basidiomycota	92	0	0
Fungi incertae sedis	32	12	12
Blastocladiomycota	0	0	0
Chytridiomycota	11	12	12
Cryptomycota	1	0	0
Microsporidia	7	0	0
Mucoromycota	11	0	0
Zoopagomycota	2	0	0
Bacteria	1,807	101	85
Archaea	765	104	5

10.1128/mBio.00967-21.7 DATA SET S4 Raw phylogenetic tree used in Fig. 3. File provided in Newick format. Download Data Set S4, TXT file, 0.00 MB. Copyright © 2021 Perli et al. 2021 Perli et al. https://creativecommons.org/licenses/by/4.0/ This content is distributed under the terms of the Creative Commons Attribution 4.0 International license.

The Adc bacterium-to-chytrid HGT event was further confirmed by using Abaccus, an automated phylogeny-aware and topology-based algorithm (29). Abaccus uses the topology of a given tree to determine taxonomic level “jumps” (J) and “losses” (L) between a seed sequence (*Nc*AdcA) and every other node in the tree. The tree of *Nc*AdcA orthologs resulted in a J of 4 and L of 3, meaning that the node comprising *Nc*AdcA “jumps” 4 taxonomic levels, which could only be explained by complete losses in 3 of these taxonomic levels. These J and L values obtained for the tree of *Nc*AdcA orthologs are higher than Abaccus’ default HGT cutoff values (J ≥ 2 and L ≥ 3) and are independent of the evolutionary model used to infer the tree (PROTGTR [30], JTT [31], and LG [32]).

Comparison of bacterial PanDs (Q0TLK2 from Escherichia coli and P9WIL2 from Mycobacterium tuberculosis) against Adcs from other bacteria (B8I983 from *C. cellulolyticum*) and eukaryotes (including A7U8C7 from Tribolium castaneum) showed only little sequence homology between *Nc*Adc*s*, known bacterial PanDs, and eukaryotic Adcs (Data Set S5). The only conserved region encompassed the full length of PanDs (126 to 139 amino acids), which represents less than 60% of the full length of other Adc sequences (e.g., *Nc*Adc*A* is 625 amino acids long). These sequence comparisons, together with the intron-exon structures verified with RNA-seq data (Fig. S1), show that *NcadcA* has acquired eukaryotic features while retaining homology to its bacterial ancestor, as is typical for prokaryotic genes acquired by fungal genomes (33).

10.1128/mBio.00967-21.8 DATA SET S5 Multiple sequence alignment. Selected l-aspartate decarboxylases are compared against known bacterial PanDs to show very little sequence conservation. Download Data Set S5, TXT file, 0.01 MB. Copyright © 2021 Perli et al. 2021 Perli et al. https://creativecommons.org/licenses/by/4.0/ This content is distributed under the terms of the Creative Commons Attribution 4.0 International license.

### Neocallimastigomycetes *PfnadB*, *PfnadA*, and *NcadcA* genes support aerobic pyridine nucleotide and CoA synthesis in yeast.

Neocallimastigomycetes were previously reported to have acquired an l-aspartate oxidase (*nadB*) and a quinolinate synthase (*nadA*) gene by HGT (6). Hence, UniProt entries A0A1Y1V2P1 and A0A1Y1VAT1 from Piromyces finnis were functionally reassigned as NadA and NadB candidates, and the corresponding genes were tentatively named *PfnadB* and *PfnadA*. These sequences, together with *NcadcA*, were codon optimized and tested to bypass the corresponding oxygen-requiring reactions in S. cerevisiae.

The *BNA2* and *FMS1* genes of S. cerevisiae were deleted by Cas9-mediated genome editing. The inability of strain IMK877 (*bna2*Δ) to synthesize quinolinic acid and of strain IMX2292 (*fms1*Δ) to synthesize β-alanine was evident from their inability to grow on glucose synthetic medium lacking nicotinic acid (SMDΔnic) and pantothenate (SMDΔpan), respectively (Table 2). Strain IMK877 was used for heterologous complementation studies with codon-optimized expression cassettes for *PfnadB* and *PfnadA*, while an expression cassette for *N. californiae NcadcA* (A0A1Y1ZL74) was introduced into strain IMX2292. Congenic strains expressing previously characterized *NADB* and *NADA* genes from Arabidopsis thaliana (*At*NadB and *At*NadA; Q94AY1 and Q9FGS4, respectively) (21), and a previously characterized gene from Tribolium castaneum encoding an aspartate decarboxylase (*Tc*PanD; A7U8C7) (22) were tested in parallel.

**TABLE 2:** Aerobic characterization of engineered strains

Strain	Growth rate (h^−1^)^*a*^		
	SMD	SMDΔnic	SMDΔpan
IMX585 (*FMS1 BNA2*)	0.40 ± 0.01	0.40 ± 0.02	0.11 ± 0.01
IMX2292 (*fms1*Δ)	0.39 ± 0.01		<0.01
IMX2305 (*fms1*Δ *TcPAND*)	0.39 ± 0.01		0.39 ± 0.01
IMX2300-1 (*fms1*Δ *NcadcA*)	0.34 ± 0.01		0.34 ± 0.01
IMK877 (*bna2*Δ)	0.40 ± 0.01	<0.01	
IMX2301 (*bna2*Δ *PfnadB PfnadA*)	0.37 ± 0.01	0.14 ± 0.01	
IMX2302 (*bna2*Δ *AtNADB AtNADA*)	0.40 ± 0.01	<0.01	

a Specific growth rates of S. cerevisiae strains grown in SMD, SMDΔnic, and SMDΔpan media. The values are averages and mean deviations of data from at least two independent cultures of each strain.

Aerobic growth of the engineered S. cerevisiae strains was characterized in shake-flask cultures on SMD or on either SMDΔnic or SMDΔpan (Table 2). In contrast to the reference strain IMK877 (*bna2*Δ), S. cerevisiae IMX2301 (*bna2*Δ *PfnadB PfnadA*) grew in SMDΔnic, indicating complementation of the *bna2*Δ-induced nicotinate auxotrophy by *PfnadB* and *PfnadA*. However, the specific growth rate of the engineered strain in these aerobic cultures was approximately 3-fold lower than that of the reference strain IMX585 (*BNA2*) (Table 2). Strain IMX2302 (*bna2*Δ *AtNADB AtNADA*) did not grow in SMDΔnic, suggesting that the plant NadB and/or NadA proteins were either not functionally expressed or not able to complement the nicotinate auxotrophy in these aerobic yeast cultures.

Strain IMX2300 (*fms1*Δ *NcadcA*) grew in SMDΔpan, indicating complementation of the pantothenate auxotrophy. However, this strain reproducibly showed a lag phase of approximately 48 h upon its first transfer from SMD to SMDΔpan and grew exponentially thereafter at a rate of 0.34 ± 0.01 h^−1^. To explore whether the lag phase of strain IMX2300 reflected selection of a spontaneous mutant, it was subjected to three sequential transfers in SMDΔpan. A single-colony isolate, IMX2300-1, from the adapted population showed a specific growth rate of 0.34 ± 0.01 h^−1^ in both SMD and SMDΔpan (Table 2). Whole-genome sequencing of IMX2300-1 did not reveal any mutations in coding DNA sequences that were considered physiologically relevant in this context compared to the nonadapted strain IMX2300 (BioProject accession number PRJNA634013). This observation indicated that the lag phase of strain IMX2300 most likely reflected a physiological adaptation or culture heterogeneity rather than a mutational event (34).

The specific growth rate of S. cerevisiae IMX2305 (*fms1*Δ *TcPAND*) on SMDΔpan did not significantly differ from that of the reference strain IMX585 on SMD, and it was almost 4-fold higher than the specific growth rate of the reference strain on SMDΔpan. These results are consistent with a previous study on functional expression of *TcPAND* in S. cerevisiae (35).

### Expression of Neocallimastigomycetes *PfnadB*, *PfnadA*, and *NcadcA* suffices to enable anaerobic pyridine nucleotide and CoA synthesis in yeast.

**FIG 4:**
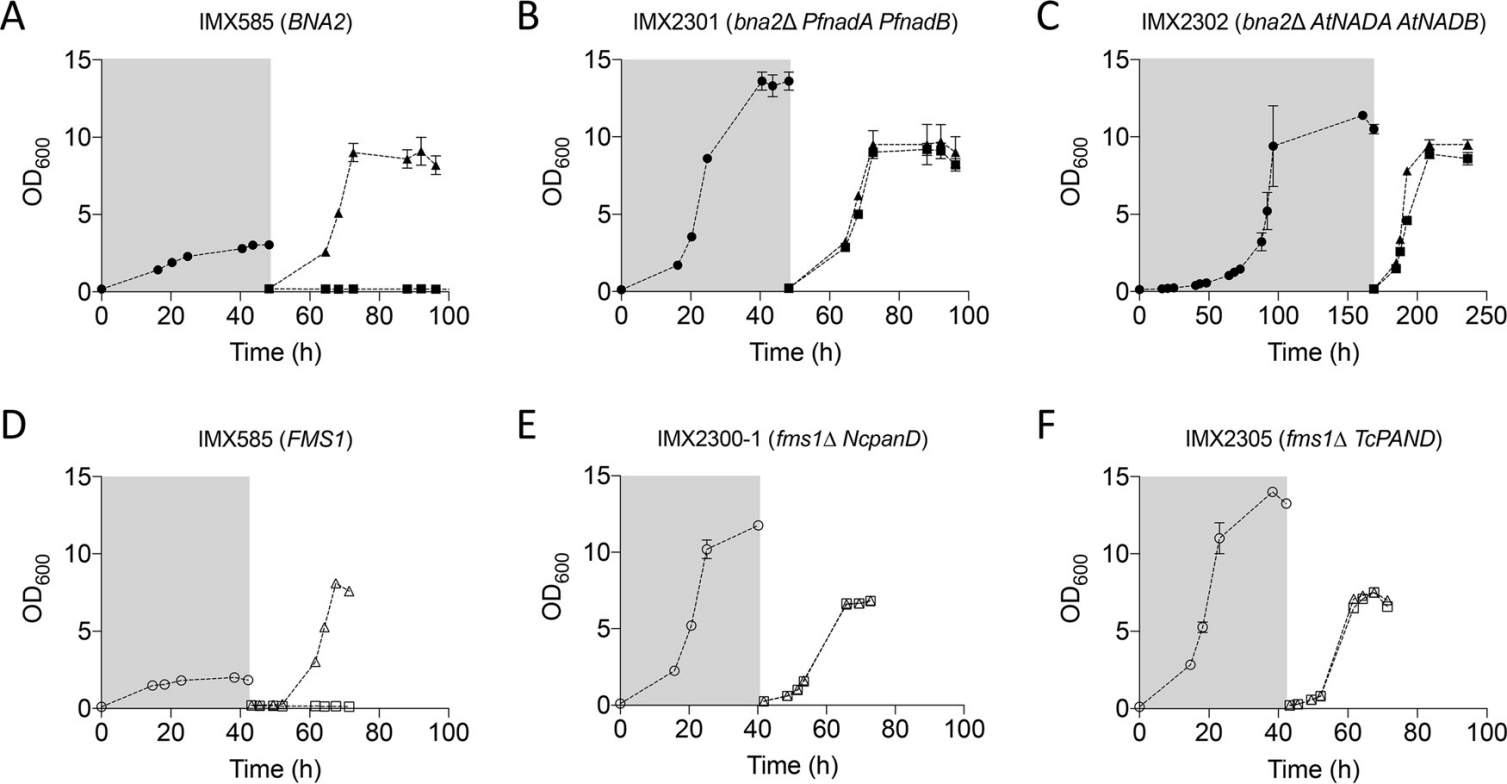
Anaerobic growth of S. cerevisiae strains dependent or independent on supplementation of nicotinic acid (NA) or pantothenic acid (PA) in SMD medium containing Tween 80 and ergosterol. Strains IMX585 (A), IMX2301 (*bna2*Δ *PfnadB PfnadA*) (B), and IMX2302 (*bna2*Δ *AtNADB AtNADA*) (C) transferred to medium with 2% glucose with (▴) or without (■) nicotinate after a carry-over phase in SMDΔnic containing 4% glucose (● in gray box). Strains IMX585 (D), IMX2300-1 (*fms1*Δ *NcadcA*) (E), and IMX2305 (*fms1*Δ *TcPAND*) (F) transferred to medium with (△) or without (□) pantothenate after a carry-over phase in SMDΔpan containing 4% glucose (○ in gray box). Anaerobic conditions in the chamber were maintained using a palladium catalyst and a 5% hydrogen concentration. Error bars represent the mean deviations from independent cultures (*n* = 2).

To investigate whether expression of heterologous *PfnadB* and *PfnadA* and that of *NcadcA* were sufficient to enable anaerobic growth in the absence of nicotinate and pantothenate, respectively, growth of the engineered S. cerevisiae strains on SMD, SMDΔnic, and/or SMDΔpan was monitored in an anaerobic chamber (Fig. 4).

Growth experiments on SMDΔnic or SMDΔpan were preceded by a cultivation cycle on the same medium, supplemented with 50 g liter^−1^ instead of 20 g liter^−1^ of glucose to ensure complete depletion of any surplus cellular contents of pyridine nucleotides, CoA, or relevant intermediates. Indeed, upon a subsequent transfer to SMDΔnic or SMDΔpan, the reference strain IMX585 (*BNA2 FMS1*), expressing the native oxygen-dependent pathways for nicotinate and β-alanine synthesis, showed no growth (Fig. 4A, B, and C).

Both engineered strains IMX2301 (*bna2*Δ *PfnadB PfnadA*) and IMX2302 (*bna2*Δ *AtNADB AtNADA*) grew anaerobically on SMDΔnic. This provided a marked contrast with the aerobic growth studies on this medium, in which strain IMX2302 did not grow. Strains IMX2305 (*fms1*Δ *TcPAND*) and the aerobically preadapted IMX2300-1 (*fms1*Δ *NcadcA*) both grew on SMDΔpan under anaerobic conditions (Fig. 4D, E, and F).

### Characterization of engineered yeast strains in anaerobic batch bioreactors.

The anaerobic chamber experiments did not allow quantitative analysis of growth and product formation. Therefore, growth of the S. cerevisiae strains expressing the Neocallimastigomycetes genes, IMX2301 (*bna2*Δ *PfnadB PfnadA*) and IMX2300-1 (*fms1*Δ *NcadcA*), was studied in anaerobic bioreactor batch cultures on SMDΔnic or SMDΔpan and compared to growth of S. cerevisiae IMX585 (*BNA2 FMS1*) on the same media.

**FIG 5:**
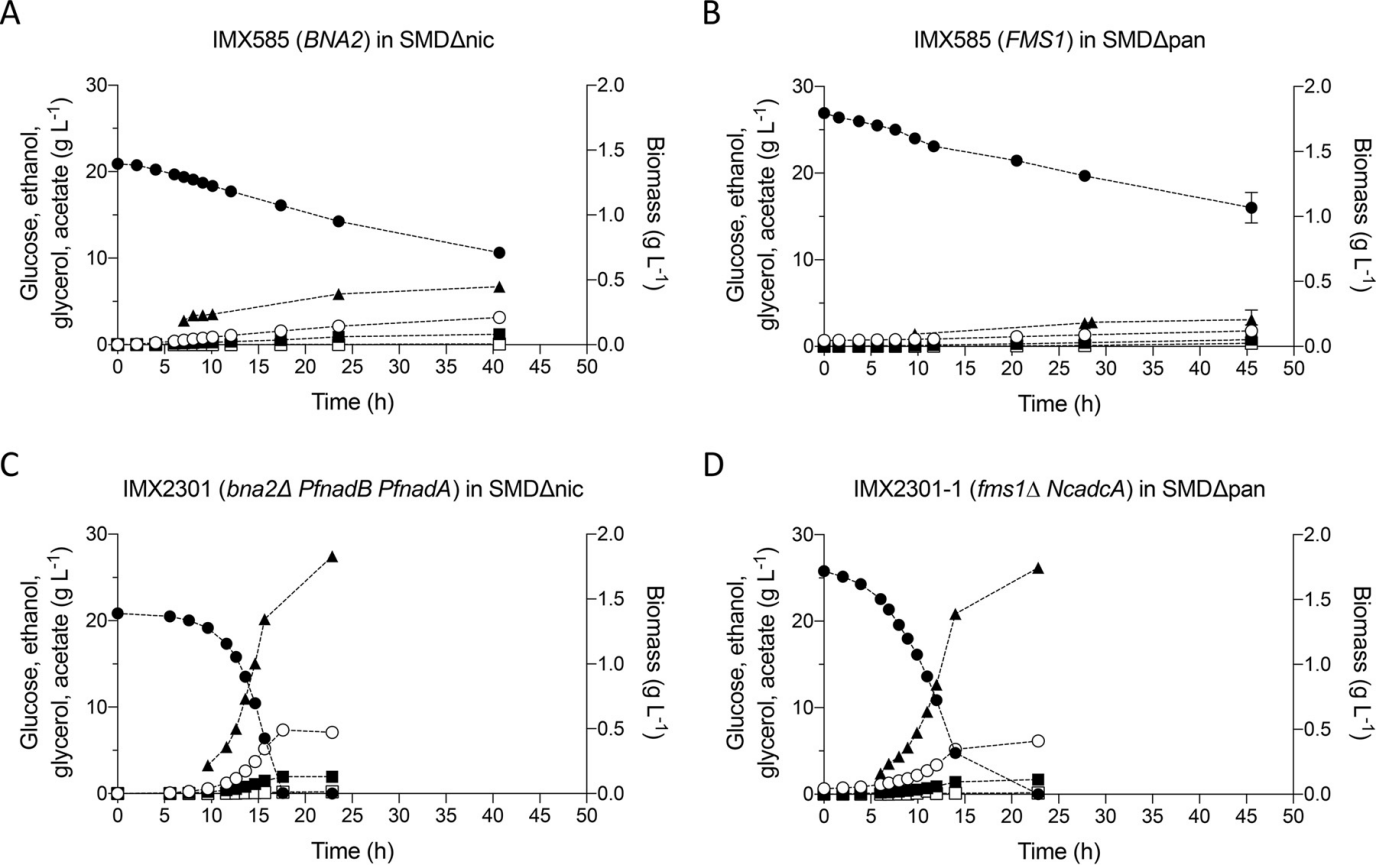
Anaerobic batch cultivation of IMX585 in SMDΔnic (A) and SMDΔpan (B), IMX2301 in SMDΔnic (C), and IMX2300-1 in SMDΔpan (D). All strains were pregrown in the corresponding medium lacking one vitamin prior to inoculation in the bioreactor to avoid carry-over effects. Values for glucose (●), ethanol (○), glycerol (■), acetate (□), and biomass (▴) are shown over time. Error bars represent the mean deviations from independent cultures (*n* = 2).

The reference strain IMX585, which typically grows fast and exponentially in anaerobic bioreactors when using complete SMD (36), exhibited extremely slow, linear growth on SMDΔnic and SMDΔpan (Fig. 5). Similar growth kinetics in “anaerobic” bioreactor cultures of S. cerevisiae on synthetic medium lacking the anaerobic growth factors Tween 80 and ergosterol were previously attributed to slow leakage of oxygen into laboratory bioreactors (37–39).

In contrast to the reference strain IMX585, the engineered strains IMX2301 and IMX2300-1 exhibited exponential anaerobic growth on SMDΔnic and SMDΔpan, respectively (Fig. 5; Table 3). The specific growth rate of strain IMX2301 (*bna2*Δ *PfnadB PfnadA*) on SMDΔnic was not significantly different from that of the reference strain on complete SMD (36), indicating full complementation of the anaerobic nicotinate auxotrophy of S. cerevisiae. The specific growth rate of strain IMX2300-1 (*fms1*Δ *NcadcA*) on SMDΔpan was only 20% lower than this benchmark (Table 3). Biomass and ethanol yields of strain IMX2301 grown in anaerobic batch cultures on SMDΔnic and strain IMX2300-1 grown on SMDΔpan were not significantly different from those of the reference strain IMX585 grown on complete SMD (*P* value > 0.05) (Table 3).

**TABLE 3:** Maximum specific growth rate (μ_max_) and yields of glycerol, biomass, and ethanol on glucose in anaerobic bioreactor batch cultures of S. cerevisiae strains IMX585, IMX2301, and IMX2300-1^*a*^

Strain	Medium	μ_max_ (h^−1^)	Yield (g g^−1^)		
			Y glycerol/glucose	Y biomass/glucose	Y ethanol/glucose
IMX585^*b*^ (*FMS1 BNA2*)	SMD	0.32 ± 0.00	0.105 ± 0.000	0.094 ± 0.004	0.372 ± 0.001
IMX2301 (*bna2*Δ *PfnadB PfnadA*)	SMDΔnic	0.31 ± 0.01	0.103 ± 0.003	0.090 ± 0.002	0.372 ± 0.002
IMX2300-1 (*fms1*Δ *NcadcA*)	SMDΔpan	0.25 ± 0.00	0.104 ± 0.000	0.081 ± 0.001	0.364 ± 0.003

a Cultures were grown on SMD, SMDΔnic, or SMDΔpan with 20 g liter^−1^ glucose as the carbon source (pH 5). Growth rates and yields were calculated from the exponential growth phase. The ethanol yield was corrected for evaporation. Values represent averages and mean deviations of data from independent cultures (*n* = 2). Carbon recovery in all fermentations was between 95% and 100%.

b Data from reference 36.

## DISCUSSION

This study shows how oxygen-independent pantothenate and nicotinate prototrophy can be conferred to the facultatively anaerobic yeast S. cerevisiae by heterologous expression of *NcadcA*, *PfnadB*, and *PfnadA* genes from Neocallimastigomycetes as well as corresponding orthologs from other species (*TcPAND*, *AtNADB*, and *AtNADA*). These results also provide insights into how acquisition of these genes by HGT conferred selective advantage to Neocallimastigomycetes’ ancestors under anaerobic conditions.

Results from phylogenetic analysis of Adc sequences (Fig. 2) were consistent with an earlier report on multiple evolutionary origins and variable evolutionary rates of pyridoxal-5′-phosphate-dependent enzymes, including Adc and glutamate decarboxylases (40, 41). A separate clade of Neocallimastigomycetes sequences shows homology with characterized glutamate decarboxylases (e.g., Q04792 from S. cerevisiae and K4HXK6 from Lactobacillus brevis) (Fig. 2). These results further support acquisition of an Adc-encoding DNA sequence by HGT rather than by neofunctionalization of a glutamate decarboxylase gene.

The characterized *Nc*AdcA (A0A1Y1ZL74) yielded the highest homology with orthologous sequences from chytrid fungi and anaerobic bacteria. This observation is in agreement with previous research showing that HGT events played a major role in shaping the genomes of Neocallimastigomycetes (4, 6, 7), with *Firmicutes* and *Proteobacteria* as prominent sequence donors (6). Specifically, closer bacterial orthologs to *Nc*AdcA were found in genome sequences of *A. hydrophila* (*Proteobacteria*) and *C. cellulolyticum* (*Firmicutes*). These bacterial species are anaerobic, and considering their ecological niches (waterborne and decayed grass/ruminal fluid, respectively [24, 26]), the results agree with current hypotheses of these types of bacteria donating genes to anaerobic gut fungi and subsequently driving a mammalian transition to herbivory (6, 8). Since *Nc*AdcA orthologs were found in 5 of the 6 chytrids analyzed, the Adc HGT transfer event appears to have preceded the 66 (±10) million years ago (MYA) estimate for divergence of Neocallimastigomycetes from other chytrids (8), although this estimate may be contended by more recent phylogenomic analyses for the whole fungal kingdom (42).

*Nc*AdcA orthology and phylogenetic analyses revealed Phialocephala subalpina as the only other nonchytrid non-Neocallimastigomycetes eukaryote to have a separate Adc-like protein. This fungus is a root endophyte and was previously proposed have obtained multiple genes by HGT from bacterial donors (43). However, A0A1L7WVG4 (PAC_06602), here identified as an Adc ortholog, was not among the 21 genes of *P. subalpina* listed as likely acquired by HGT from nonfungal species. Since the phylogenetic placement of the putative *P. subalpina* Adc was close to bacterial as well as archaeal sequences, further studies are needed to reveal its evolutionary history.

Whereas an alternative to the kynurenine pathway for NAD^+^ synthesis was previously inferred from genome sequence analysis, the pathway by which Neocallimastigomycetes synthesize coenzyme A had not previously been explored. Six pathways for synthesis of the essential CoA precursor β-alanine are known: (A) decarboxylation of l-aspartate (44), (B) transamination of malonate semialdehyde with l-glutamate as amino donor (45) or l-alanine (46), (C) reduction of uracil followed by hydrolysis of the resulting dihydrouracil (47), (D) oxidative cleavage of spermine to 3-aminopropanal followed by oxidation of the aldehyde group (16), (E) 2,3-aminomutase of alanine (48), and (F) addition of ammonia to acryloyl-CoA, followed by hydrolysis of the resulting CoA thioester (48). Of these pathways, all but option D, in principle, can occur in the absence of oxygen. Yeasts and other filamentous fungi typically form β-alanine from spermine (pathway D), but in some species, the use of pathway C was also reported (49).

While the aspartate decarboxylation route (A) has not previously been demonstrated in wild-type fungi, functional expression of bacterial and *T. castaneum Tc*PanD was used in metabolic engineering of S. cerevisiae to boost supply of β-alanine as a precursor for 3-hydroxypropionate production (22, 35). Wild-type S. cerevisiae strains cannot grow in anaerobic environments unless supplemented with pantothenate. Expression of either *NcadcA* or *TcPAND* in an *fms1*Δ S. cerevisiae strain, which lacks the native oxygen-dependent pantothenate biosynthesis pathway, enabled growth in pantothenate-free medium under aerobic and anaerobic conditions. Although the different specific growth rates of S. cerevisiae strains expressing *NcadcA* or *TcPAND* indicate that changing expression levels and/or origin of ADC-encoding genes may be required to achieve optimal growth, these results provide a proof of principle for a simple metabolic engineering strategy to eliminate oxygen requirements for pantothenate synthesis.

Genomic analyses previously suggested that genomes of Neocallimastigomycetes encode a putative l-aspartate oxidase (NadB) and quinolinate synthase (NadA) as alternatives to the canonical kynurenine pathway found in other fungi (6). Additionally, Neocallimastigomycetes appear to have acquired both *nadB* and *nadA* through HGT (6). Until now, functionality of these Neocallimastigomycetes proteins in an oxygen-independent pathway for synthesis of quinolinate from l-aspartate had not been demonstrated.

Our results demonstrate that expression of *nadB* and *nadA* homologs, either from the Neocallimastigomycetes *P. finnis* or from the plant *A. thaliana* (21), suffice to allow anaerobic synthesis of NAD^+^ of S. cerevisiae. Due to the involvement of the Bna2 and Bna4 oxygenases in NAD^+^ synthesis by S. cerevisiae, nicotinate is an essential growth factor for this yeast under anaerobic conditions (14, 50, 51). A similar strategy was recently successfully applied to enable oxygen-independent synthesis of pyridine nucleotides in the bacterium Pseudomonas putida (52). The present study represents the first demonstration of a metabolic engineering strategy to eliminate oxygen requirements for NAD^+^ synthesis in a yeast.

Functional expression of heterologous NadA quinolinate synthases in S. cerevisiae was observed despite the fact that these enzymes are [4Fe-4S] iron-sulfur cluster proteins (53, 54), which are notoriously difficult to functionally express in the yeast cytosol (55–58). However, earlier studies on functional expression of the [4Fe-4S] activating protein of bacterial pyruvate-formate lyase (59, 60) demonstrated that low-levels of expression can occur without modification of the yeast machinery for cytosolic assembly of [Fe-S] clusters. The inability of *At*NadB and *At*NadA to support NAD^+^ synthesis in aerobic cultures may be due to oxygen sensitivity of the [4Fe-4S] cluster in the *At*NadA quinolinate synthase domain (61). In contrast to *Pf*NadA, *At*NadA carries an N-terminal SufE domain which, in other organisms, has been demonstrated to allow this oxygen-sensitive enzyme to remain active under aerobic conditions by reconstituting its [Fe-S] cluster (61).

This work contributes to the understanding of how Neocallimastigomycetes adapted to their anaerobic lifestyle by acquiring genes that enable oxygen-independent synthesis of central metabolic cofactors. Experiments with engineered S. cerevisiae strains showed that contribution of the heterologous genes to *in vivo* oxygen-independent cofactor synthesis did not require additional mutations in the host genome. These results indicate how acquisition of functional genes by HGT, even if their expression was initially suboptimal, could have conferred an immediate advantage to ancestors of anaerobic fungi living in cofactor-limited anoxic environments. A similar approach was recently applied to study the physiological impact on S. cerevisiae of expressing a heterologous gene encoding squalene-tetrahymanol cyclase, which in Neocallimastigomycetes, produces the sterol surrogate tetrahymanol (13). Functional analysis by heterologous expression in S. cerevisiae circumvents the current lack of tools for genetic modification of Neocallimastigomycetes (2) and can complement biochemical studies (3–5) and genome sequence analyses (6, 7).

Pantothenate and nicotinate, together with the other compounds belonging to the B-group of water-soluble vitamins, are standard ingredients of chemically defined media for aerobic and anaerobic cultivation of yeasts (62). S. cerevisiae strains have been shown to contain the genetic information required for *de novo* synthesis of these vitamins and can even be experimentally evolved for complete prototrophy for individual vitamins by prolonged cultivation in single-vitamin-depleted media (63, 64). In large-scale processes, addition of nutritional supplements increases costs, reduces shelf-life of media, and increases the risk of contamination during their storage (62). Therefore, metabolic engineering strategies for enabling oxygen-independent synthesis of NAD^+^ and pantothenate are of particular interest for the development robust yeast strains with minimal nutritional requirements that can be applied in anaerobic biofuel production (62). Further studies of the unique evolutionary adaptations of Neocallimastigomycetes may well provide additional inspiration for engineering robust fungal cell factories that operate under anaerobic conditions.

## MATERIALS AND METHODS

### Homology and phylogenetic analyses.

A set of 51 amino acid sequences previously used to discriminate between l-aspartate decarboxylases (Adc) and glutamate decarboxylases (23) was reused to identify candidate Neocallimastigomycetes Adc sequences. These sequences were used as queries against a database containing all 58,109 Neocallimastigomycetes proteins deposited in UniProt trembl (Release 2019_02), which represented 5 species (Neocallimastix californiae, Anaeromyces robustus, *Piromyces* sp. E2, Piromyces finnis, and Pecoramyces ruminantium), and extracted according to the NCBI taxonomic identifier (taxid) 451455. Sequence homology was analyzed using BLASTP 2.6.0+ (65) with 10^−6^ as the E value cutoff, resulting in 16 Neocallimastigomycetes sequences as shared hits from all 51 queries (see Table S1 in the supplemental material). Four of these sequences showing homology to experimentally characterized Adc proteins originated from *N. californiae* and were checked for RNA-seq read coverage and splicing junction support, revealing A0A1Y1ZL74 as the best candidate (Fig. S1). For this purpose, Illumina libraries were obtained from the Sequence Read Archive using accession SRR7140690 (66) which were then mapped using STAR 2.6.1a_08-27 (67) against genome assembly GCA_002104975. Alignments were processed using SAMtools 1.3.1 (68) and visualized using Artemis (69).

A0A1Y1ZL74, also referred to as *Nc*AdcA, was used for a second round of homology search using HMMER 3.2 (70) against 3 different databases built from UniProt release 2019_02 to include all RefSeq sequences from *Bacteria* (taxid 2), Eukarya (taxid 2759), and Archaea (taxid 2157; TrEMBL and Swiss-Prot categories were also included in this case). Selection for hits with more than 60% alignment length over the query sequence and an E value of <10^−6^ resulted in a total of 325 sequences (103 from *Bacteria*, 101 from Eukaryota, and 121 from Archaea) (Data Set S1).

The set of 325 A0A1Y1ZL74 homologous sequences, together with those from Tomita et al. (23), and the 16 Neocallimastigomycetes sequences from that described above were used for further phylogenetic analyses. A total of 387 sequences (Data Set S2) were aligned with MAFFT v7.402 (71) in “einsi” mode, and alignments were trimmed with trimAl v1.2 (72) in “gappyout” mode and then used to build a maximum likelihood phylogenetic tree with RAxML-NG 0.8.1 (72) using default parameters, with the exception of the use of the PROTGTR+FO model and 100 bootstrap replicates. The resulting phylogenetic tree drawn with iTOL (73) is shown in Fig. 2, and corresponding sequences and the unannotated tree are provided in Data Sets S2 and S3.

Proteomes from species showing an Adc homolog were extracted into individual fasta files and used for (co)orthology search with ProteinOrtho6 (74). A0A1Y1ZL74 ortholog groups were then extracted and subjected to alignment, trimming, and phylogenetic analysis as described above. The resulting phylogenetic tree is shown in Fig. 3, and corresponding sequences and the unannotated tree are provided in Data Sets S2 and S4.

Abaccus v1.1 (29) (https://github.com/Gabaldonlab/Abaccus) was used to search the tree presented in Fig. 3 (Data Set S4) for evidence of HGT. For this purpose, the taxonomy table provided as default was supplemented with definitions for the additional chytrids considered in this study.

Multiple-sequence alignment was also performed with Clustal omega 1.2.4 (75) to compare selected amino acid sequences showing candidate and experimentally characterized Adcs against bacterial PanDs. These sequences and alignments are shown in Data Set S5.

### Strains, media, and maintenance.

S. cerevisiae strains used and constructed in this study (Table 4) were derived from the CEN.PK lineage (76). Yeast cultures were routinely propagated in YP (10 g liter^−1^ Bacto yeast extract [Becton, Dickinson and Co., Sparks, MD], 20 g liter^−1^ Bacto peptone [Becton, Dickinson and Co.]) or synthetic medium (SM) (77). YP and SM were autoclaved at 121°C for 20 min. SM was then supplemented with 1 ml liter^−1^ of filter-sterilized vitamin solution (0.05 g liter^−1^ d-(+)-biotin, 1.0 g liter^−1^ d-calcium pantothenate, 1.0 g liter^−1^ nicotinic acid, 25 g liter^−1^ *myo*-inositol, 1.0 g liter^−1^ thiamine hydrochloride, 1.0 g liter^−1^ pyridoxol hydrochloride, 0.20 g liter^−1^ 4-aminobenzoic acid). Where indicated, nicotinic acid or pantothenic acid was omitted from the vitamin solution, yielding SM without nicotinic acid (SMΔnic) and SM without pantothenic acid (SMΔpan), respectively. A concentrated glucose solution was autoclaved separately for 15 min at 110°C and added to SM and YP to a concentration of 20 g liter^−1^ or 50 g liter^−1^, yielding SMD and YPD, respectively. SMD with urea or acetamide instead of ammonium sulfate (SMD-urea and SMD-Ac, respectively) was prepared as described previously (78, 79). For anaerobic growth experiments, sterile media were supplemented with Tween 80 (polyethylene glycol sorbate monooleate; Merck, Darmstadt, Germany) and ergosterol (≥95% pure; Sigma-Aldrich, St. Louis, MO) as described previously (39). Yeast strains were grown in 500-ml shake flasks containing 100 ml medium or in 100-ml shake flasks containing 20 ml medium. Shake-flask cultures were incubated at 30°C and shaken at 200 rpm in an Innova Incubator (Brunswick Scientific, Edison, NJ). Solid media were prepared by adding 15 g liter^−1^ Bacto agar (Becton, Dickinson and Co.) and, when indicated, 200 mg liter^−1^ G418 (Thermo Scientific, Waltham, MA). After genotyping, engineered strains were restreaked twice to select single clones. Removal of the guide RNA (gRNA)-carrying plasmid was conducted as previously described (80). Stock cultures were prepared by adding glycerol to a final concentration of 33% (vol/vol), frozen, and stored at −80°C.

**TABLE 4:** S. cerevisiae strains used in this study

Name	Relevant genotype^*a*^	Parental strain	Reference
CEN.PK113-7D	*MAT***a** *URA3*		76
CEN.PK113-5D	*MAT***a** *ura3-52*		76
IMX585	*MAT***a** *can1*Δ::*Spycas9*-natNT2 *URA3*	CEN.PK113-7D	80
IMX581	*MAT***a** *ura3-52 can1*Δ::*Spycas9*-natNT2	CEN.PK113-5D	80
IMX2292	*MAT***a** *can1*Δ::*Spycas9*-natNT2 *URA3 fms1*Δ	IMX585	63
IMK877	*MAT***a** *can1*Δ::*Spycas9*-natNT2 *URA3 bna2*Δ	IMX585	This study
IMX2301	*MAT***a** *can1*Δ::*Spycas9*-natNT2 *URA3 bna2*Δ *sga1*::p*TDH3*-*PfnadA*-t*ENO1* p*CCW12*-*PfnadB*-t*ENO2*	IMK877	This study
IMX2302	*MAT***a** *can1*Δ::*Spycas9*-natNT2 *URA3 bna2*Δ *sga1*::p*TDH3*-*AtNADA*-t*ENO1* p*CCW12-AtNADB*-t*ENO2*	IMK877	This study
IMX2293	*MAT***a** *ura3-52 can1*Δ::*Spycas9*-natNT2 *fms1*Δ	IMX581	This study
IMX2300	*MAT***a** *ura3-52*::p*TDH3-NcadcA*-t*ENO2 URA3 can1*Δ::*Spycas9*-natNT2 *fms1*Δ	IMX2293	This study
IMX2300-1	*MAT***a** *ura3-52*::p*TDH3-NcadcA*-t*ENO2 URA3 can1*Δ::*Spycas9*-natNT2 *fms1*Δ Colony isolate 1	IMX2300	This study
IMX2305	*MAT***a** *ura3-52*::p*RPL12b-TcPAND*-t*TDH1 URA3 can1*Δ::*Spycas9*-natNT2 *fms1*Δ	IMX2293	This study

a *Spy*, Streptococcus pyogenes; *Pf*, *Piromyces finnis*; *Nc*, *Neocallimastix californiae*; *At*, Arabidopsis thaliana; *Tc*, Tribolium castaneum.

### Molecular biology techniques.

DNA was PCR amplified with Phusion Hot Start II high-fidelity polymerase (Thermo Scientific) and desalted or PAGE-purified oligonucleotide primers (Sigma-Aldrich) according to the manufacturers’ instructions. DreamTaq polymerase (Thermo Scientific) was used for diagnostic PCR. Oligonucleotide primers used in this study are listed in Table 5. PCR products were separated by gel electrophoresis using 1% (wt/vol) agarose gel (Thermo Scientific) in Tris-acetate-EDTA (TAE) buffer (Thermo Scientific) at 100 V for 25 min and purified with either GenElute PCR clean-up kit (Sigma-Aldrich) or with Zymoclean gel DNA recovery kit (Zymo Research, Irvine, CA). Plasmids were purified from E. coli using a Sigma GenElute plasmid kit (Sigma-Aldrich). Yeast genomic DNA was isolated with the SDS-lithium acetate (LiAc) protocol (81). Yeast strains were transformed with the lithium acetate method (82). Four to eight single colonies were restreaked three consecutive times on selective media, and diagnostic PCRs were performed to verify their genotypes. Escherichia coli XL1-Blue was used for chemical transformation (83). Plasmids were then isolated and verified by either restriction analysis or by diagnostic PCR. Lysogeny broth (LB; 10 g liter^−1^ Bacto tryptone, 5 g liter^−1^, Bacto yeast extract with 5 g liter^−1^ NaCl) was used to propagate E. coli XL1-Blue. LB medium was supplemented with 100 mg liter^−1^ ampicillin for selection of transformants. The overnight-grown bacterial cultures were stocked by adding sterile glycerol at a final concentration of 33% (vol/vol), after which samples were frozen and stored at −80°C.

**TABLE 5:** Oligonucleotide primers used in this study

Primer no.	Primer sequence	Product(s)^*a*^
6005	GATCATTTATCTTTCACTGCGGAGAAG	gRNA pROS plasmid backbone amplification
11861	TGCGCATGTTTCGGCGTTCGAAACTTCTCCGCAGTGAAAGATAAATGATCCAGAAGAGCATATTCCATTTGTTTTAGAGCTAGAAATAGCAAGTTAAAATAAG	2-μm fragment for *BNA2* gRNA plasmid
11862	GTCAACGCCGATATGAACAACACTTCCATAACCGGACCACAAGTACTACATAGAACAAAACATTATACATTTTATTAACGCCCCCCCTTTTTTTTTTTTGTTTGATGCAGAAGCCTCGCA	*BNA2* KO repair oligonucleotide fwd
11863	TGCGAGGCTTCTGCATCAAACAAAAAAAAAAAAGGGGGGGCGTTAATAAAATGTATAATGTTTTGTTCTATGTAGTACTTGTGGTCCGGTTATGGAAGTGTTGTTCATATCGGCGTTGAC	*BNA2* KO repair oligonucleotide rev
11877	GCATCGTCTCATCGGTCTCATGCATCGTCTCATCGGTCTCAT	YTKflank_TcPanD_fwd
11878	ATGCCGTCTCAGGTCTCAGGATTCACAAATCGGAACCCAAT	YTKflank_TcPanD_rev
16721	CAATTCGTCGCAATACAACGCAGTTCGAGTTTATCATTATCAATACTGC	*pTDH3* amplification_fwd
16722	ATGGTTTCTTTGTCGACCATTTTGTTTGTTTATGTGTGTTTATTCGA	*pTDH3* amplification_rev
16723	AACACACATAAACAAACAAAATGGTCGACAAAGAAACCATTAA	*NcPanD* amplification_fwd
16724	AATTCTTAGTTAAAAGCACTTTACTTGATCAGCTTGTGGTTCA	*NcPanD* amplification_rev
16725	ACCACAAGCTGATCAAGTAAAGTGCTTTTAACTAAGAATTATTAGTCTTTTCTG	*tENO2* amplification_fwd
16726	CTGACGAGCAGATTTCCAGCATTTTTCAAACTGCAAATTCAAGAA	*tENO2* amplification_rev
16727	GAATTTGCAGTTTGAAAAATGCTGGAAATCTGCTCGTCAG	pYTK096 amplification_fwd
16728	ATAATGATAAACTCGAACTGCGTTGTATTGCGACGAATTG	pYTK096 amplification_rev
13527	AACAAGAAGTGAGTTAATAAAGGCAAAAACAGTGGTCGTGTGAGAAGTAGAATTTCACCTAGACGTGGAATCTATTTTTTCGAAATTACTTACACTTTTGACGGCTAGAAAAG	*FMS1* KO repair oligonucleotide fwd
13528	CTTTTCTAGCCGTCAAAAGTGTAAGTAATTTCGAAAAAATAGATTCCACGTCTAGGTGAAATTCTACTTCTCACACGACCACTGTTTTTGCCTTTATTAACTCACTTCTTGTT	*FMS1* KO repair oligonucleotide rev
13123	TTTACAATATAGTGATAATCGTGGACTAGAGCAAGATTTCAAATAAGTAACAGCAGCAAACAGTTCGAGTTTATCATTATCAATACTG	*NadA* repair fragment fwd
13124	ATAGCATAGGTGCAAGGCTCTCGCCGCTTGTCGAGCTATTGGCATGGATGTGCTCCCTAAATACATGGGTGACCAAAAGAGC	*NadA* repair fragment rev
13125	TTAGGGAGCACATCCATGCCAATAGCTCGACAAGCGGCGAGAGCCTTGCACCTATGCTATCACCCATGAACCACACGG	*NadB* repair fragment fwd
10710	TATATTTGATGTAAATATCTAGGAAATACACTTGTGTATACTTCTCGCTTTTCTTTTATTATTTTTCAAACTGCAAATTCAAGAAAAAGCCAC	NadB repair fragment rev

a fwd, forward; rev, reverse.

### Plasmid construction.

Plasmids used and cloned in this study are shown in Table 6. Plasmids carrying two copies of the same gRNA were cloned by Gibson assembly (80, 84). In brief, an oligonucleotide carrying the gene-specific 20-bp target sequence and a homology flank to the plasmid backbone was used to amplify the fragment carrying the 2-μm origin of replication sequence by using pROS13 as the template. The backbone linear fragment was amplified using primer 6005 and pROS11 as the template (85). The two fragments were then gel purified and assembled *in vitro* using the NEBuilder HiFi DNA assembly master mix (New England BioLabs, Ipswich, MA) according to the manufacturer’s instructions. Transformants were selected on LB plates supplemented with 100 mg liter^−1^ ampicillin or 50 mg liter^−1^ kanamycin. Primer 11861 was used to amplify the 2-μm fragment containing two identical gRNA sequences for targeting *BNA2*. The PCR product was then cloned in a pROS11 backbone yielding plasmid pUDR315.

**TABLE 6:** Plasmids used in this study

Name	Characteristics^*a*^	Reference or source
pROS10	2-μm *bla* ori *URA3* gRNA-*CAN1.*Y gRNA-*ADE2*.Y	80
pROS11	2-μm *bla* ori *amdSYM* gRNA-*CAN1*.Y gRNA-*ADE2.*Y	80
pROS13	2-μm *bla* ori *kanMX* gRNA-*CAN1.*Y gRNA-*ADE2.Y*	80
pUDR119	2-μm *bla* ori *amdSYM* gRNA-*SGA1* gRNA-*SGA1*	93
pYTK009	p*TDH3 cat* ColE1	86
pYTK010	p*CCW12 cat* ColE1	86
pYTK017	p*RPL18B cat* ColE1	86
pYTK051	t*ENO1 cat* ColE1	86
pYTK055	t*ENO2 cat* ColE1	86
pYTK056	t*TDH1 cat* ColE1	86
pYTK096	ConLS′ *gfp* ConRE′*URA3 ntpII* ColE1 5′*URA3*	86
pGGKd017	ConLS′ *gfp* ConRE′ *URA3* 2 μm *bla* ColE1	94
pCfB-361	2-μm *bla* ori pTEF1-*TcPAND*^*b*^*-tCYC1 HIS3*	35
pUDR652	*bla* 2-μm *amdSYM* gRNA-*FMS1* gRNA-*FMS1*	63
pUD652	*bla PfnadA*^*b*^	GeneArt, this study
pUD653	*bla PfnadB*^*b*^	GeneArt, this study
pUD1095	*bla NcadcA*^*b*^	GeneArt, this study
pUD1096	*bla AtNADA*^*b*^	GeneArt, this study
pUD1097	*nptII AtNADB*^*b*^	GeneArt, this study
pUDR315	*bla* 2-μm *amdSYM* gRNA-*BNA2* gRNA-*BNA2*	This study
pUDI168^*c*^	p*RPL18B*-*TcPAND*^*b*^-t*TDH1 URA3 ntpII* ColE1 5′*URA3*	This study
pUDI242	p*TDH3*-*NcadcA*^*b*^-t*ENO2 URA3 ntpII* ColE1 5′*URA3*	This study
pUDI243^*d*^	p*TDH3*-*PfNADA*^*b*^-t*ENO1 URA3 ntpII* ColE1 5′*URA3*	This study
pUDI244^*e*^	p*CCW12*-*PfnadB*^*b*^-t*ENO2 URA3 ntpII* ColE1 5′*URA3*	This study
pUDI245^*f*^	p*TDH3*-*AtNADA*^*b*^-t*ENO1 URA3 ntpII* ColE1 5′*URA3*	This study
pUDE931^*g*^	p*CCW12*-*AtNADB*^*b*^-t*ENO2 URA3* 2-μm *bla* ColE1	This study

a *Pf*, *Piromyces finnis*; *Nc*, *Neocallimastix californiae*; *At*, Arabidopsis thaliana; *Tc*, Tribolium castaneum.

b Codon optimized for expression in S. cerevisiae.

c Result of the Golden gate assembly of plasmids pYTK017, TcPAND PCR, pYTK056, and pYTK096.

d Result of the Golden gate assembly of plasmids pYTK009, pUD652, pYTK051, and pYTK096.

e Result of the Golden gate assembly of plasmids pYTK010, pUD653, pYTK055, and pYTK096.

f Result of the Golden gate assembly of plasmids pYTK009, pUD1096, pYTK051, and pYTK096.

g Result of the Golden gate assembly of plasmids pYTK010, pUD1097, pYTK055, and pGGKd017.

The coding sequences for *AtNADA*, *AtNADB*, *PfnadA*, *PfnadB*, and *NcadcA* were codon optimized for expression in S. cerevisiae and ordered as synthetic DNA through GeneArt (Thermo Fisher Scientific). The plasmids carrying the expression cassettes for *TcPAND*, *AtNADA*, *AtNADB*, *PfnadA*, and *PfnadB* were cloned by Golden Gate assembly using the Yeast Toolkit (YTK) DNA parts (86). These plasmids were cloned using the pYTK096 integrative backbone that carries long homology arms to the *URA3* locus and a *URA3* expression cassette allowing for selection on SM lacking uracil. The *TcPAND* coding sequence was amplified using the primer pair 11877/11878 and pCfB-361 as the template. Then, the linear *TcPAND* gene and plasmids pUD1096, pUD1097, pUD652, and pUD653 carrying the coding sequences for *AtNADA*, *AtNADB*, *PfnadA*, and *PfnadB*, respectively, were combined together with YTK-compatible part plasmids in BsaI (New England BioLabs) Golden Gate reactions to yield plasmids pUDI168, pUDI245, pUDE931, pUDI243, and pUDI244, respectively.

The plasmid carrying the expression cassette for *NcadcA* was cloned by Gibson assembly. The *pTDH3* promoter, the *NcadcA* coding sequence, the *tENO2* terminator, and the pYTK0096 backbone were amplified by PCR using primer pairs 16721/16722, 16723/16724, 16725/16726, and 16727/16728, respectively, using pYTK009, pUD1095, pYTK055, and pYTK096 as the template, respectively. Each PCR product was then gel purified and combined in equimolar amounts in a Gibson reaction that yielded pUDI242.

### Strain construction.

S. cerevisiae strains were transformed using the LiAc–single-stranded DNA (ssDNA)–polyethylene glycol (PEG) and CRISPR/Cas9 method (80, 82, 87). For deletion of the *BNA2* gene, IMX585 (*can1*Δ::*Spycas9-natNT2*) was transformed with 500 ng of the *BNA2*-targeting gRNA plasmid pUDR315 together with 500 ng of the annealed primer pair 11862/11863 as the repair double-stranded DNA (dsDNA) oligonucleotide, yielding strain IMK877. The resulting strain was then used for the integration of the two heterologous *NADB-A* pathways. Expression cassettes for *AtNADA*, *AtNADB*, *PfnadA*, and *PfnadB* were amplified from plasmids pUDI245, pUDE931, pUDI243, and pUDI244, respectively, using primer pairs 13123/13124, 13125/10710, 13123/13124, and 13125/10710, respectively. Then, 500 ng of each pair of gel purified repair cassettes was cotransformed in IMK877 together with 500 ng of the *SGA1*-targeting gRNA plasmid, yielding IMX2302 (*sga1*::*AtNADA AtNADB*) and IMX2301 (*sga1*::*PfnadA PfnadB*).

For deletion of the *FMS1* gene, IMX581 (*can1*Δ::S*pycas9-natNT2 ura3-52*) was transformed with 500 ng of the *FMS1*-targeting gRNA plasmid pUDR652 together with 500 ng of the annealed primer pair 13527/13528 as the repair dsDNA oligonucleotide, resulting in IMX2293. Then, 500 ng each of plasmids pUDI168 and pUDI242 carrying the expression cassettes for *TcPAND* and *NcadcA*, respectively, were digested with NotI (Thermo Fisher) and separately transformed in IMX2293, yielding IMX2305 and IMX2300, respectively. Selection of IMX2305 and IMX2300 was performed on an SMD agar plate, since the integration of each Adc-encoding cassette also restored the *URA3* phenotype. In contrast, selection of IMK877 was conducted on SMD-Ac agar plates, while selection of IMX2302, IMX2301, and IMX2293 was conducted on YPD-G418 agar plates. Strains IMK877, IMX2300, IMX2302, and IMX2301 were stocked in SMD, while IMX2305 and IMX2293 were stocked in SMDΔpan and YPD, respectively.

### Aerobic growth studies in shake flasks.

For the determination of the specific growth rate of the engineered strains under aerobic conditions, a frozen aliquot was thawed and used to inoculate a 20-ml wake-up culture that was then used to inoculate a preculture in a 100-ml flask. The exponentially growing preculture was then used to inoculate a third flask to an initial optical density at 600 nm (OD_660_) of 0.2. The flasks were then incubated, and growth was monitored using a 7200 Jenway Spectrometer (Jenway, Stone, United Kingdom). Specific growth rates were calculated from at least five time points in the exponential growth phase of each culture. Wake-up and precultures of IMX2301 and IMX2302 were grown in SMDΔnic. Wake-up and precultures of IMX2300 and IMX2305 were grown in SMDΔpan, while wake-up and precultures of IMK877 and IMX2292 were grown in SMD.

### Anaerobic growth studies in shake flasks.

Anaerobic shake-flask based experiments were performed in a Lab Bactron 300 anaerobic workstation (Sheldon Manufacturing Inc., Cornelius, OR) containing an atmosphere of 85% N_2_, 10% CO_2_, and 5% H_2_. Flat-bottom shake flasks of 50 ml were filled with 40 ml SMD-urea medium containing 50 g liter^−1^ glucose as the carbon source to ensure depletion of the vitamin/growth factor of interest and 20 g liter^−1^ glucose for the first transfer. Media were supplemented with vitamins, with and without pantothenic acid or nicotinic acid as indicated, and in all cases, supplemented with Tween 80 and ergosterol. Sterile medium was placed inside the anaerobic chamber 24 h prior to inoculation for removal of oxygen. Traces of oxygen were continuously removed with a regularly regenerated Pd catalyst for H_2_-dependent oxygen removal placed inside the anaerobic chamber. Aerobic overnight shake-flask cultures on SMD-urea were used to inoculate the anaerobic shake flask without pantothenic acid or without nicotinic acid at an initial OD_600_ of 0.2. Cultures were cultivated at 30°C with continuous stirring at 240 rpm on an IKA KS 260 Basic orbital shaker platform (Dijkstra Verenigde BV, Lelystad, the Netherlands). Periodic optical density measurements at a wavelength of 600 nm using an Ultrospec 10 cell density meter (Biochrom, Cambridge, United Kingdom) inside the anaerobic environment were used to follow the growth over time. After growth had ceased and the OD_600_ no longer increased, the cultures were transferred to SMD-urea with 20 g liter^−1^ glucose at an OD_600_ of 0.2 (39).

### Anaerobic bioreactor cultivation.

Anaerobic bioreactor batch cultivation was performed in 2-liter laboratory bioreactors (Applikon, Schiedam, the Netherlands) with a working volume of 1.2 liters. Bioreactors were tested for gas leakage by applying 30 kPa overpressure while completely submerging them in water before autoclaving. Anaerobic conditions were maintained by continuous sparging of the bioreactor cultures with 500 ml N_2_ min^−1^ (≤0.5 ppm O_2_, HiQ nitrogen 6.0; Linde Gas Benelux, Schiedam, the Netherlands). Oxygen diffusion was minimized by using Fluran tubing (14 Barrer O_2_, F-5500-A; Saint-Gobain, Courbevoie, France) and Viton O-rings (Eriks, Alkmaar, the Netherlands). Bioreactor cultures were grown on either SMDΔpan or SMDΔnic with ammonium sulfate as the nitrogen source. pH was controlled at 5 using 2 M KOH. The autoclaved mineral salts solution was supplemented with 0.2 g liter^−1^ sterile antifoam emulsion C (Sigma-Aldrich). Bioreactors were continuously stirred at 800 rpm, and temperature was controlled at 30°C. Evaporation of water and volatile metabolites was minimized by cooling the outlet gas of bioreactors to 4°C in a condenser. The outlet gas was then dried with a PermaPure PD-50T-12MPP dryer (Permapure, Lakewood, NJ) prior to analysis. CO_2_ concentrations in the outlet gas were measured with an NGA 2000 Rosemount gas analyzer (Emerson, St. Louis, MO). The gas analyzer was calibrated with reference gas containing 3.03% CO_2_ and N6-grade N_2_ (Linde Gas Benelux).

Frozen glycerol stock cultures were used to inoculate aerobic 100-ml shake-flask cultures on either SMDΔpan or SMDΔnic. Once the cultures reached and OD_660_ of >5, a second 100-ml aerobic shake-flask preculture on the same medium was inoculated. When this second preculture reached the exponential growth phase, biomass was harvested by centrifugation at 3,000 × *g* for 5 min and washed with sterile demineralized water. The resulting cell suspension was used to inoculate anaerobic bioreactors at an OD_660_ of 0.2.

### Analytical methods.

Biomass dry weight measurements of the bioreactor batch experiments were performed using preweighed nitrocellulose filters (0.45 μm; Gelman Laboratory, Ann Arbor, MI). Ten-milliliter culture samples were filtrated, and then the filters were washed with demineralized water prior to drying in a microwave oven (20 min at 360 W) and weight measurement. Metabolite concentrations in culture supernatants were analyzed by high-performance liquid chromatography (HPLC). In brief, culture supernatants were loaded on an Agilent 1260 HPLC system (Agilent Technologies, Santa Clara, CA) fitted with a Bio-Rad HPX 87 H column (Bio-Rad, Hercules, CA). The flow rate was set at 0.6 ml min^−1^, and 0.5 g liter^−1^ H_2_SO_4_ was used as the eluent. An Agilent refractive-index detector and an Agilent 1260 variable wavelength detector (VWD) were used to detect culture metabolites (88). An evaporation constant of 0.008 divided by the volume in liters was used to correct HPLC measurements of ethanol in the culture supernatants, taking into account changes in volume caused by sampling (89). Statistical analysis on product yields was performed by means of an unpaired two-tailed Welch’s *t* test.

### Whole-genome sequencing and analysis.

Genomic DNA of strains IMX2300 and IMX2300-1 was isolated with a Blood & Cell Culture DNA kit with 100/G Genomics-tips (Qiagen, Hilden, Germany) according to the manufacturer’s instructions. The MiSeq reagent kit v3 (Illumina, San Diego, CA) was used to obtain 300-bp reads for paired-end sequencing. Genomic DNA was sheared to an average of 550-bp fragments using an M220 ultrasonicator (Covaris, Woburn, MA). Libraries were prepared by using a TruSeq DNA PCR-free library preparation kit (Illumina) according to the manufacturer’s instructions. The samples were quantified by quantitative PCR (qPCR) on a Rotor-Gene Q PCR cycler (Qiagen) using the Collibri library quantification kit (Invitrogen, Carlsbad, CA). Finally, the library was sequenced using an Illumina MiSeq sequencer (Illumina), resulting in a minimum 50-fold read coverage. Sequenced reads were mapped using BWA 0.7.15-r1142-dirty (90) against the CEN.PK113-7D genome (91) containing an extra contig with the relevant integration cassette. Alignments were processed using SAMtools 1.3.1 (68), and sequence variants were called using Pilon 1.18 (92), processed with ReduceVCF 12 (https://github.com/AbeelLab/genometools/blob/master/scala/abeel/genometools/reducevcf/ReduceVCF.scala) and annotated using VCFannotator (http://vcfannotator.sourceforge.net/) against GenBank accession GCA_002571405.2 (62).

### Data availability.

DNA sequencing data of the Saccharomyces cerevisiae strains IMX2300 and IMX2300-1 were deposited at NCBI (https://www.ncbi.nlm.nih.gov/) under BioProject accession number PRJNA634013. All measurement data and calculations used to prepare Fig. 4 and 5 and Tables 2 and 3 of the manuscript are available at the 4TU.Centre for research data repository (https://researchdata.4tu.nl/) at https://doi.org/10.4121/uuid:c3d2326d-9ddb-469a-b889-d05a09be7d97.
