# Supplementary material for: Identification of Oxygen-Independent Pathways for Pyridine Nucleotide and Coenzyme A Synthesis in Anaerobic Fungi by Expression of Candidate Genes in Yeast
Source: mBio. 2021 Jun 22;12(3):e00967-21. doi: 10.1128/mBio.00967-21 (PMC8262920; doi:10.1128/mBio.00967-21)
Supplement: TABLE S1 [file mbio.00967-21-st001.docx]

### **Supplementary Table S1: Neocallimastigomycete BLASTP hits**. Obtained using the dataset from Tomita et al. as queries against a Neocallimastigomycete-specific amino acid sequence database, and an e-value < 10-6 as cutoff.

| **Entry** | **Entry name** | **Status** | **Protein names** | **Gene names** | **Organism** | **Length** | **Cross-reference (Pfam)** |
| --- | --- | --- | --- | --- | --- | --- | --- |
| A0A1Y1VBC9 | A0A1Y1VBC9_9FUNG | unreviewed | Cysteine desulfurase | BCR36DRAFT_287428 | Piromyces finnis | 415 | PF00266; |
| A0A1Y1VIL9 | A0A1Y1VIL9_9FUNG | unreviewed | PLP-dependent transferase | BCR36DRAFT_345242 | Piromyces finnis | 600 | PF00282; |
| A0A1Y1WT12 | A0A1Y1WT12_9FUNG | unreviewed | Glutamate decarboxylase (EC 4.1.1.15) | BCR32DRAFT_296089 | Anaeromyces robustus | 797 | PF00282; |
| A0A1Y1X726 | A0A1Y1X726_9FUNG | unreviewed | Cysteine desulfurase | BCR32DRAFT_232915 | Anaeromyces robustus | 454 | PF00266; |
| A0A1Y1XDI3 | A0A1Y1XDI3_9FUNG | unreviewed | PLP-dependent transferase | BCR32DRAFT_266694 | Anaeromyces robustus | 594 | PF00282; |
| A0A1Y1ZL74 | A0A1Y1ZL74_9FUNG | unreviewed | PLP-dependent transferase | LY90DRAFT_708822 | Neocallimastix californiae | 625 | PF00282; |
| A0A1Y2AA19 | A0A1Y2AA19_9FUNG | unreviewed | PLP-dependent transferase (Fragment) | LY90DRAFT_677094 | Neocallimastix californiae | 433 | PF00282; |
| A0A1Y2ADH1 | A0A1Y2ADH1_9FUNG | unreviewed | PLP-dependent transferase | LY90DRAFT_463975 | Neocallimastix californiae | 577 | PF00282; |
| A0A1Y2AZ59 | A0A1Y2AZ59_9FUNG | unreviewed | Glutamate decarboxylase (EC 4.1.1.15) | LY90DRAFT_628503 | Neocallimastix californiae | 481 | PF00282; |
| A0A1Y2B2H7 | A0A1Y2B2H7_9FUNG | unreviewed | PLP-dependent transferase | LY90DRAFT_460911 | Neocallimastix californiae | 524 | PF00282; |
| A0A1Y2DBU7 | A0A1Y2DBU7_9FUNG | unreviewed | Mitochondrial cysteine desulfurase | LY90DRAFT_523242 | Neocallimastix californiae | 396 | PF00266; |
| A0A1Y3MYE4 | A0A1Y3MYE4_PIRSE | unreviewed | Uncharacterized protein | PIROE2DRAFT_17257 | Piromyces sp. (strain E2) | 450 | PF00282; |
| A0A1Y3NC87 | A0A1Y3NC87_PIRSE | unreviewed | Uncharacterized protein | PIROE2DRAFT_7587 | Piromyces sp. (strain E2) | 248 | PF00282; |
| A0A1Y3NC92 | A0A1Y3NC92_PIRSE | unreviewed | Uncharacterized protein | PIROE2DRAFT_7586 | Piromyces sp. (strain E2) | 169 |  |
| A0A1Y3NFH0 | A0A1Y3NFH0_PIRSE | unreviewed | Aminotran_5 domain-containing protein | PIROE2DRAFT_61071 | Piromyces sp. (strain E2) | 196 | PF00266; |
| A0A1Y3NIR5 | A0A1Y3NIR5_PIRSE | unreviewed | Uncharacterized protein | PIROE2DRAFT_58808 | Piromyces sp. (strain E2) | 127 | PF00282; |
