# Supplementary figures and images for: Identification of Oxygen-Independent Pathways for Pyridine Nucleotide and Coenzyme A Synthesis in Anaerobic Fungi by Expression of Candidate Genes in Yeast
Source: mBio. 2021 Jun 22;12(3):e00967-21. doi: 10.1128/mBio.00967-21 (PMC8262920; doi:10.1128/mBio.00967-21)

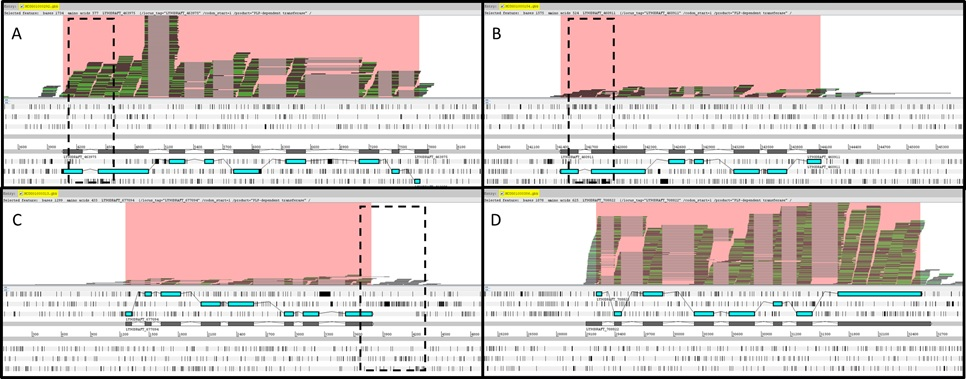

Supplement: FIG S1 [file mbio.00967-21-sf001.tif]

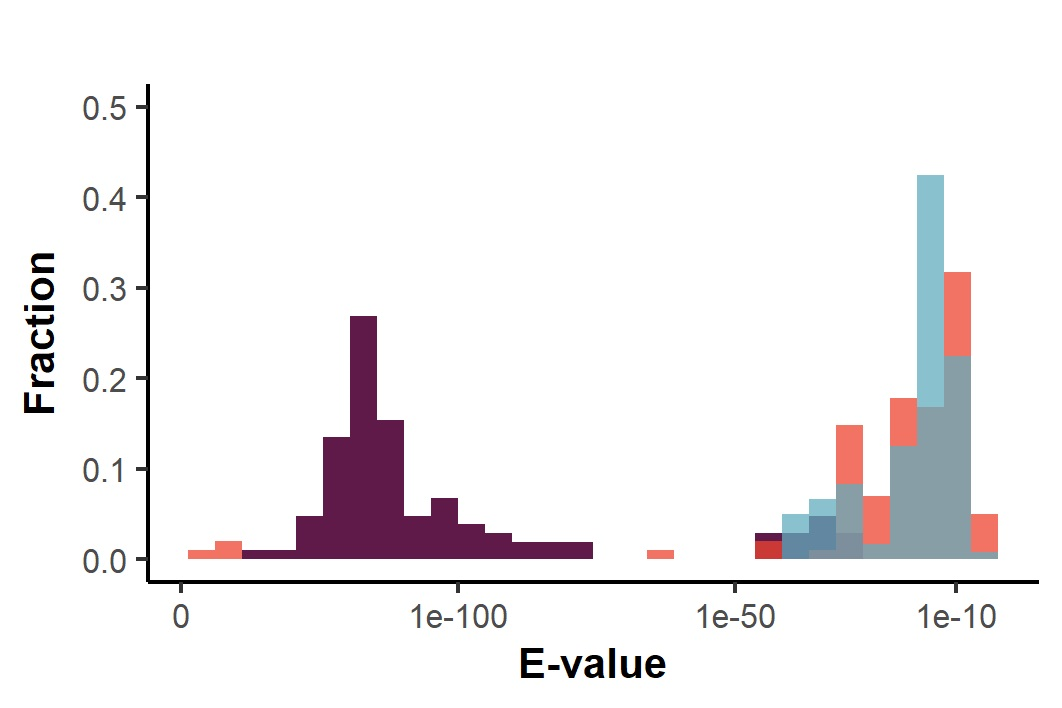

Supplement: FIG S2 [file mbio.00967-21-sf002.tif]
